# AGE-ASSOCIATED CHALLENGES AMONG HIV POPULATION IN VIETNAM: PERSPECTIVES FROM MULTIPLE STAKEHOLDERS

**DOI:** 10.1093/geroni/igad104.3778

**Published:** 2023-12-21

**Authors:** Qing Xia, Yuan Xiong, Fei Sun, Giang Le, Van Hoang, Khanh Nguyen, Huong Dang, Ethan Liu

**Affiliations:** Michigan State University, East Lansing, Michigan, United States; Michigan State University, East Lansing, Michigan, United States; Michigan State University, East Lansing, Michigan, United States; Hanoi Medical University, Hanoi, An Giang, Vietnam; Hanoi Medical University, Hanoi, An Giang, Vietnam; Hanoi Medical University, Hanoi, An Giang, Vietnam; Hanoi Medical University, Hanoi, An Giang, Vietnam; Phillips Exeter Academy, Exeter, New Hampshire, United States

## Abstract

HIV remains a significant global public health concern. The growing population of aging individuals living with HIV in Vietnam presents a multifaceted pressing issue, calling for research, policy, and practice interventions. This study aims to identify the aging-associated healthcare needs among the HIV population from three stakeholder groups: healthcare professionals, family caregivers, and persons living with HIV-AIDs. Qualitative data was collected through five focus group interviews involving 10 people living with HIV (PLWH), 9 caregivers, and 8 healthcare providers in Hanoi, Vietnam, in March 2023. Data analysis followed thematic analysis techniques, uncovering recurring patterns and themes across the three participant groups.PLWH reflected diverse experiences, showing mental health struggles, aging-related concerns, and a lack of preparation due to health challenges or unexpected longevity. Resilience and optimism are also manifested in PLWH. Caregivers demonstrated crucial roles, including bridging care, medication adherence, and financial and emotional support. They highlighted the necessity of training programs to enhance their care capabilities. Both PLWH and caregivers expressed a desire for formalized training programs on HIV and aging. Furthermore, healthcare providers reported mental and physical health changes among aging PLWH, such as insomnia, forgetfulness, and memory deterioration. They noted a lack of resources and called for comprehensive training across medical staff to enhance the well-being of the aging PLWH.This study demonstrated the complicated age-associated needs among PLWH in Vietnam. The findings underscore the need for policy and practice innovations to address aging-related cognitive, mental health, and long-term care concerns.

